# A collagen-based microwell migration assay to study NK-target cell interactions

**DOI:** 10.1038/s41598-019-46958-3

**Published:** 2019-07-23

**Authors:** Per E. Olofsson, Ludwig Brandt, Klas E. G. Magnusson, Thomas Frisk, Joakim Jaldén, Björn Önfelt

**Affiliations:** 1 Division of Biophysics, Department of Applied Physics, Science for Life Laboratory, KTH Royal Institute of Technology, Tomtebodavägen 23 A, 171 65 Stockholm, Sweden; 2 Department of Signal Processing, ACCESS Linnaeus Centre, KTH Royal Institute of Technology, Stockholm, Sweden; 3 Department of Microbiology, Tumor and Cell Biology, Karolinska Institute, Solna, Sweden

**Keywords:** Innate lymphoid cells, Microfluidics, Biological physics

## Abstract

Natural killer (NK) cell cytotoxicity in tissue is dependent on the ability of NK cells to migrate through the extracellular matrix (ECM) microenvironment. Traditional imaging studies of NK cell migration and cytotoxicity have utilized 2D surfaces, which do not properly reproduce the structural and mechanical cues that shape the migratory response of NK cells *in vivo*. Here, we have combined a microwell assay that allows long-term imaging and tracking of small, well-defined populations of NK cells with an interstitial ECM-like matrix. The assay allows for long-term imaging of NK–target cell interactions within a confined 3D volume. We found marked differences in motility between individual cells with a small fraction of the cells moving slowly and being confined to a small volume within the matrix, while other cells moved more freely. A majority of NK cells also exhibited transient variation in their motility, alternating between periods of migration arrest and movement. The assay could be used as a complement to *in vivo* imaging to study human NK cell heterogeneity in migration and cytotoxicity.

## Introduction

Natural killer (NK) cells, a class of innate lymphoid cells (ILCs), are large granular lymphocytes that have the capacity to kill virus-infected and transformed cells. NK cell cytotoxicity is regulated by integrating the combined input of signaling from activating and inhibitory receptors clustered at the immune synapse (IS)^1^. The main inhibitory signaling is provided by killer-cell immunoglobulin-like receptors (KIRs), which interact with cognate HLA class I molecules^2^. If KIRs lack cognate HLA in *trans*, the inhibitory signal is absent and cytotoxicity is not inhibited, making the NK cell more likely to trigger directed secretion of cytotoxic granules. Release of cytolytic granules at the IS causes the rapid incorporation of pro-apoptotic granzymes into the target cell, subsequently killing it^3^.

Cell migration is a fundamental ability underlying embryogenesis, immune function and wound healing. It is a highly complex, tightly regulated process involving systems of cell surface receptors, intracellular signaling pathways, the cytoskeleton, and it is highly dependent on interactions with the local extracellular microenvironment. Dysregulated cell migration is a hallmark of cancer, for example in the metastatic spread of solid tumors mediated by epithelial-to-mesenchymal transition^4^.

The function of NK cells depends critically on their ability to extravasate from the circulation and migrate through the extracellular matrix (ECM) toward target tissues. The ECM is the non-cellular part of tissues and forms complex, interlinked networks of crosslinked macromolecules that support tissue and modulate cell behavior. ECM composition varies between different tissues in order to fill different functions. Although the composition of the ECM varies between different species, sequencing has revealed that mammals share a conserved “matrisome” of around 300 proteins^5^.

It has long been recognized that in order to obtain more biologically relevant *in vitro* assays, we must move away from traditional 2-dimensional (2D) culture methods and approach systems that more closely resemble the actual biology in 3D. By introducing synthetic or naturally derived ECM materials, it is possible to perform experiments in more naturalistic microenvironments. Cell migration behaviors have been characterized in different types of matrices, e.g. cell-derived matrix, collagen I hydrogels, fibrin, and basement membrane extract (BME)^6^. Collagen-based matrices are among the most well-studied 3D systems. Collagen-based assays have been in use for the study of lymphocyte migration for several decades^7,8^, and protocols for imaging and analysis of cell migration in 3D collagen matrices have been established^9,10^. A collagen-based assay where adherent target cells grown at the bottom of a culture plate were overlaid with a 3D collagen matrix containing T cells has been used to study the cytotoxic behavior of T cells^11^. A recent report also showed a novel platform for studying in T cells’ interactions with dendritic cells in collagen matrixes^12^. ECM gels like collagen I and Matrigel have modest light scattering properties compared to tissue enabling optical imaging^13^.

Here we have extended a previously developed 2D microchip-based assay for studying migration and cytotoxicity to include the third spatial dimension^14^. Half-millimeter-sized wells were filled with a collagen matrix containing NK cells and target cells and the cells were followed for 9 hours assessing interactions between cells for duration and outcome. By using microwells, the same population of cells could be studied throughout the assay. State-of-the art confocal imaging gave rapid and long-term volumetric imaging by combining fast scan-speed with sensitive detection reducing phototoxicity and photobleaching. We used a recently developed software for automatic tracking of individual cells in 3D^15,16^. By allowing controlled environments and the use of human cells, this method complements current *in vivo* methods for assessing immune cell migration and contact dynamics^17,18^.

## Results

### The microchip platform

**Figure 1:**
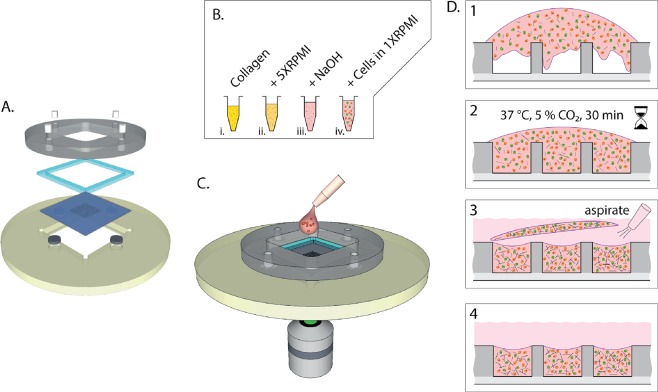
Schematic figure of experimental setup and hydrogel embedding procedure. (**A**) Exploded view of the microchip platform consisting of plastic holder with embedded stainless-steel discs, microchip, gasket, and plastic lid with embedded magnets (**B**) Procedure for preparing collagen-embedded cells mixtures. Stock solution of collagen monomers dissolved in acetic acid (i) was brought to the right concentration by addition of concentrated cell medium (ii) and reconstituted by adding NaOH (iii) to which a mixture of NK cells and target cells suspended in RPMI was added (iv). (**C**) The cell-collagen mix was rapidly deposited onto the microwell chip inserted in the assembled holder. (**D**) Schematic view of the deposit and maturation of the collagen matrix in the microwells. The viscous collagen-cell mixture was poured into the wells (1) and then incubated under physiological conditions for 30 min (2). When the matrix had set, cell medium was gently streamed over the wells which caused excess matrix to detach from the chip (3) leaving only cell-collagen mixture in the wells (4).

The microchip platform (Fig. 1A) has been described in previous publications^19–21^. It consists of a silicon-glass microchip where an array of square wells (sides 450 μm and depth 300 μm) have been etched through a silicon wafer before anodic bonding of the glass that constitutes the bottom of the wells. The microchip sits in a plastic or metallic holder made to fit on the motorized stage of an inverted microscope. Directly on top if the chip, a gasket made of polydimethylsiloxane (PDMS) prevents leakage of cell medium from the reservoir that is created when the plastic (poly methyl methacrylate) lid is clamped on top of the holder. The holder-chip-gasket-lid sandwich is secured by four neodymium magnets mounted in the lid.

Before loading the chip, NK cells and target cells were embedded in type I collagen hydrogel (Fig. 1B). The mix was then deposited onto the microwell chip (Fig. 1C) where it poured into the wells before the gel was set (Fig. 1D). After incubation (30 min, 37 °C), cell medium was gently pipetted into the reservoir from the side of the wells with the pipette tilted creating a fluid flow from the side. This caused excess gel matrix to detach from the chip so that it could be aspirated with the pipette leaving only collagen-embedded cells inside the wells and not at the top.

To ensure the robustness of our embedding procedure, NK and target cells and the matrix structure were characterized by confocal microscopy. The content in individual wells was visualized by acquiring fluorescence images from Calcein orange-labeled NK cells and Calcein green-labeled K562 target cells from 36–39 optical sections (Supporting Fig. S1A). The distribution of cells throughout the volume could be estimated by assessing the fluorescence intensity corresponding to NK and target cells in each optical section of the well. The cells were evenly distributed as indicated by diagonal lines in a plot of cumulative fluorescence versus wells depth (Supporting Fig. S1B).

To characterize the structure of the collagen matrix, we used backscattered light (reflection) from the collagen fibers. Reflection images acquired at different optical planes away from the bottom showed that the matrix had similar structure deep into the volume (Supporting Fig. S1C). It is not straightforward to make accurate estimations of pore sizes based on reflective images^22^, but a qualitative assessment indicated that it agreed with a previous estimation of ≈ 3 μm reported in the literature^23^. Thus, these results showed that NK and target cells were distributed in a homogeneous collagen mesh throughout the volume of the wells.

### NK cells display varied migration dynamics

**Figure 2:**
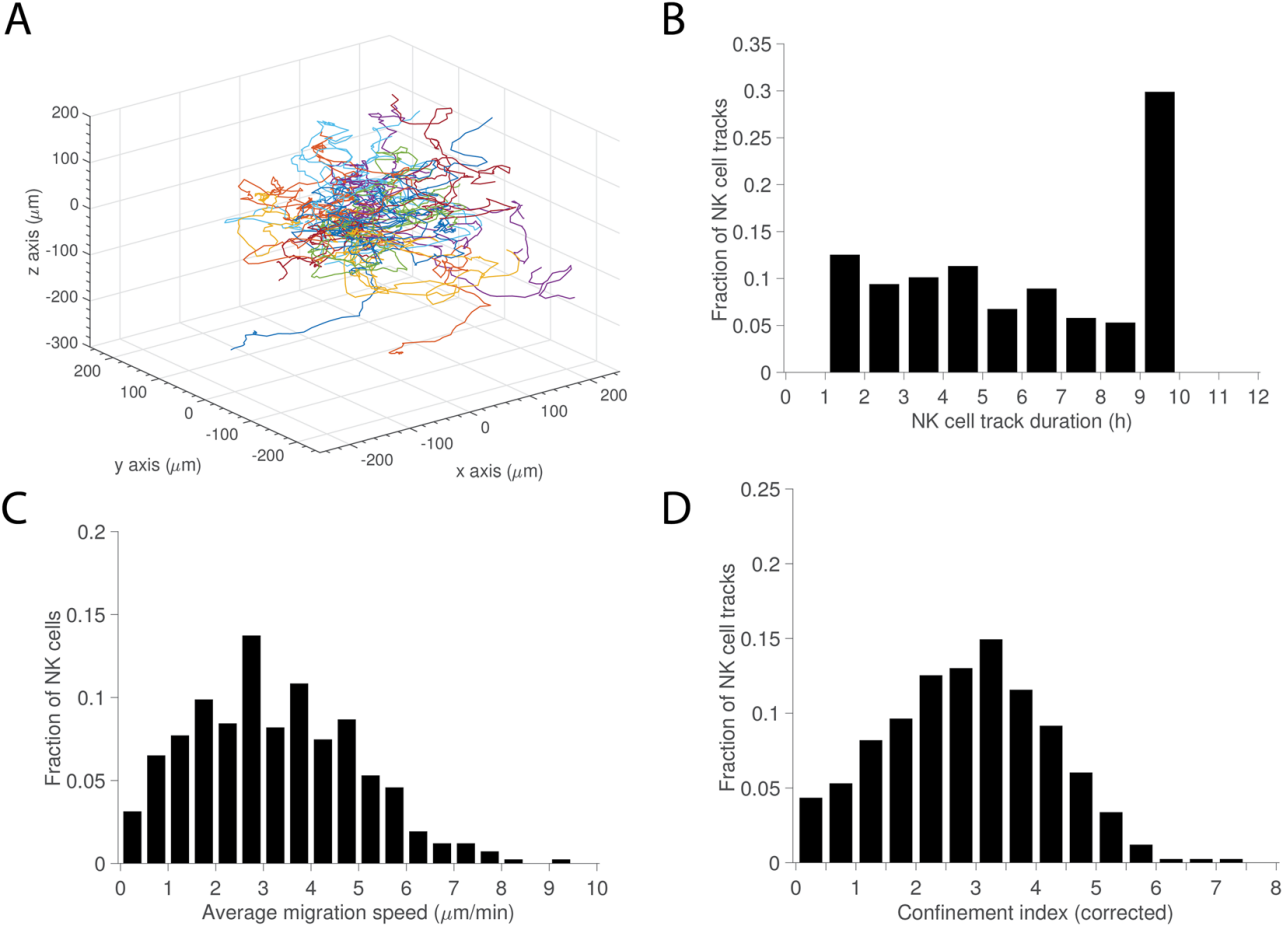
NK cell migration in 3D. (**A**) Track plot of NK cell migration trajectories centered at their respective starting points. (**B**) Distribution of NK cell track durations (>1 h). (**C**) Distribution of average migration speeds calculated from individual tracks. (**D**) Corrected confinement index calculated from individual tracks. Total number of NK cells *n* = 411.

NK and target cells embedded in collagen were imaged by time-lapse confocal microscopy acquiring a z-stack of each well every 2 minutes for 9 h. Individual NK cells were tracked in 3D by using a recently developed software, the Baxter algorithms^15,16^. A track plot of NK cell trajectories is shown in Fig. 2A and Supporting Movie 1 shows a time-sequence where tracks from four individual NK cells are shown. The algorithm detected tracks ranging from the whole duration of the experiment to 1 hour, which was set as the minimum duration of tracks to be considered for analysis (Fig. 2B). From the tracks the mean migration speed of individual NK cells was measured giving the ensemble a distribution ranging from 0.3–9.4 µm/min with an average of 3.2 ± 1.7 µm/min (Fig. 2C). This is faster compared to the speeds measured for resting (un-activated) (1.6 ± 0.6 µm/min) and IL-2 activated (1.7 ± 0.7 µm/min) human NK cells in 2D acquired with similar imaging interval^24^. Human T cells coincubated with dendritic cells loaded with either high or low affinity peptides in a collagen matrix have been found to move with speeds of 3 or 12 µm/min, respectively (sampling interval 30–60 sec)^12^. *In vivo* imaging of murine NK cells in tumors have shown NK cell speeds of about 5 µm/min in tumors (sampling interval 30 sec)^25^ and 7–10 µm/min in lymph nodes (sampling interval 18–21 sec)^26,27^. To evaluate if fluorescence labelling and repeated imaging affected the migration properties we compared our results with unlabeled NK cells imaged with minimal illumination in a conventional widefield microscope (Supporting Fig. S2). We found that unlabeled cells had a slightly higher mean migration speed, but no statistically significant difference was found. The spread in the distribution of speeds was mirrored by a similar distribution in confinement for individual NK cells (Fig. 2D).

A similar migration analysis for the K562 target cells showed that these cells were very immotile and had a high confinement index (Supporting Fig. S3). Thus, the NK cells showed individual variation in their migration behavior while the target cells were more homogenous with less motility.

### NK cells display transient changes in migration dynamics

A hallmark of lymphocyte migration is their stop-and-go behavior. This was also observed here with some tracks alternating confinement with periods of motility. To characterize these transient changes in motility, we used an analysis method based on a rolling window that stepped through the tracks point-by-point analyzing the mean squared displacement (MSD) from small sections (25 time points) of the tracks^14^. Thus, at each time point of the track, the ‘local’ motility properties can be assessed. We defined transient migration arrest periods (TMAPs) as periods when the motility coefficient was below 4.9 μm^2^/min. This threshold corresponds to diffusive movement of a spherical object of cell-size (diameter 8 μm). As in previous reports, periods of directed migration were assessed by estimating the curvature of the MSD^14^. Periods that were neither defined as migration arrest or directed migration were assigned to random movement.

**Figure 3:**
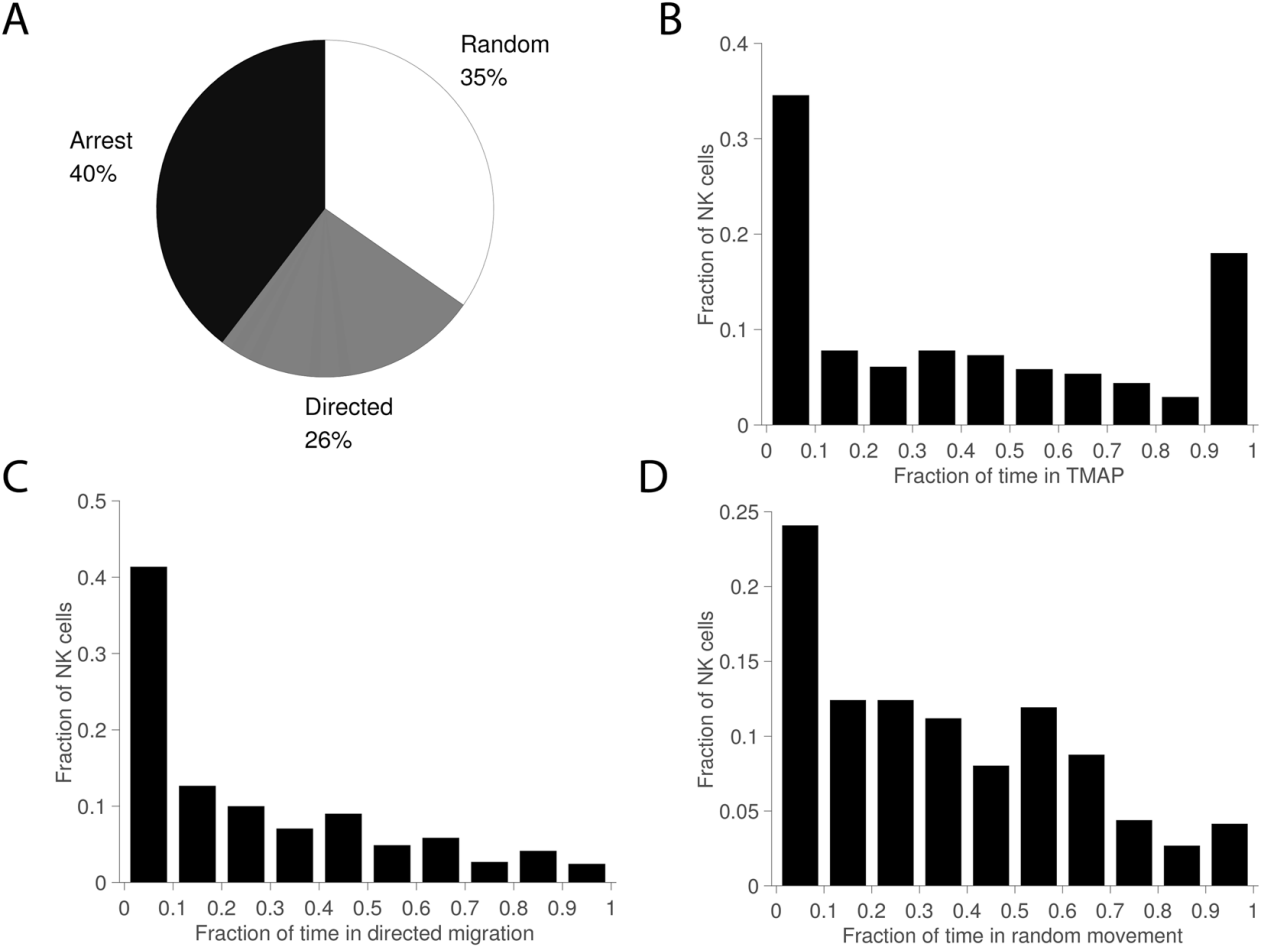
Mode of migration. (**A**) Pie chart showing distribution of time spent in transient migration arrest (TMAP), random movement, and directed migration. (**B**) Distribution of fractions of time spent in TMAP for individual tracks. (**C**) Distributions of fractions of time spent in directed migration. (**D**) Distribution of fractions of time spent in random movement. Total number of NK cells *n* = 411.

**Figure 4:**
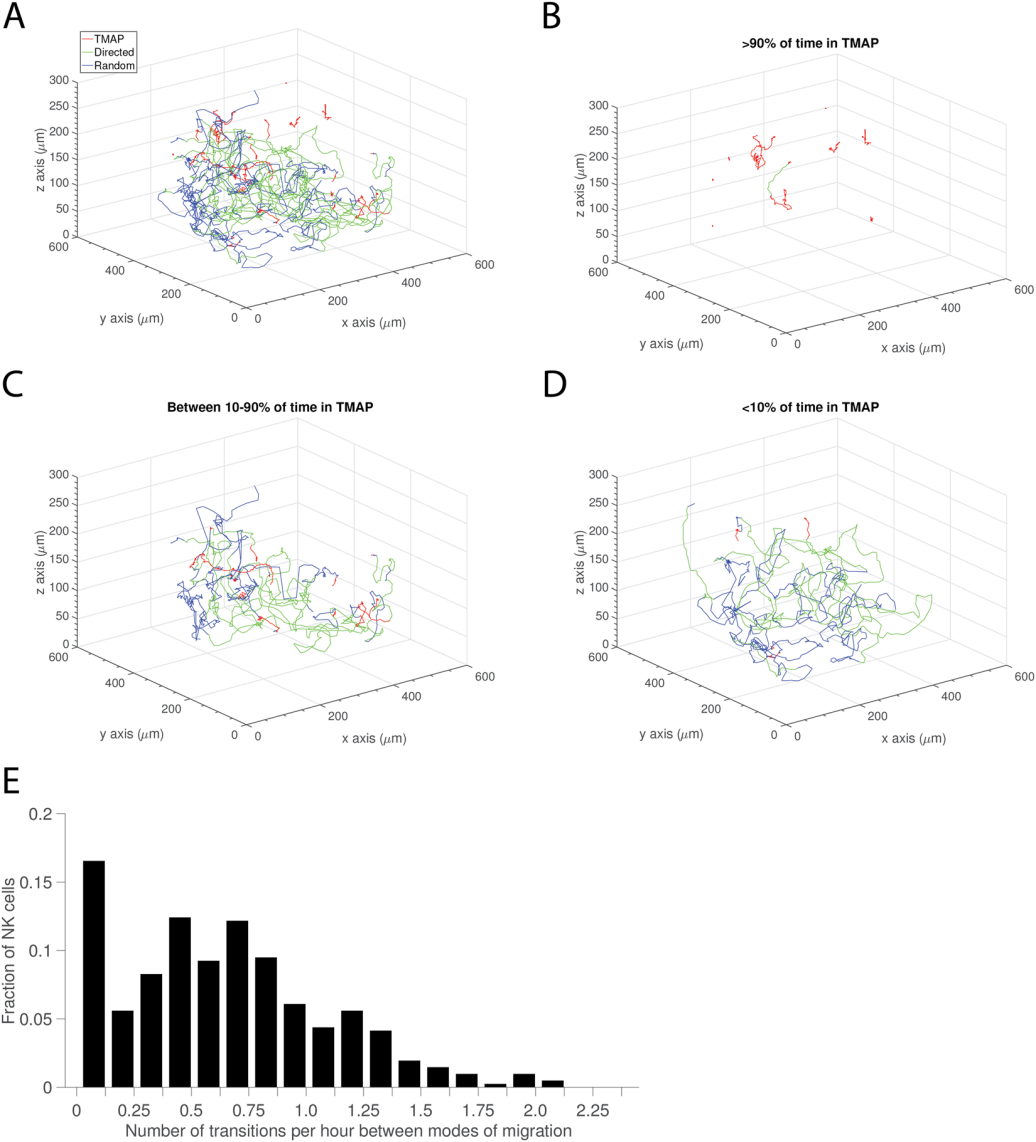
Transient migration behavior. (**A**) Track plot of NK cell migration trajectories recorded in a single well divided into modes of migration (*n* = 25). (**B**) Track plot of NK cells spending >90% of time in TMAP (*n* = 10). (**C**) Track plot of NK cells spending between 90% and 10% of time in TMAP (*n* = 9). (**D**) Track plot of NK cells spending < 10% of time in TMAP (*n* = 6). (**E**) Distribution of number of transitions between different modes of migration per hour (*n* = 411).

Overall, NK cells’ migration was relatively evenly spread across the different modes of migration with 40% of the time spent in migration arrest, 35% in random movement and 26% in directed migration (Fig. 3A). Looking at the distribution at the single cell level revealed a fraction of cells (≈18%) spending almost all their time in TMAPs (Fig. 3B). However, most of the cells were motile (random movement or directed migration) for a significant fraction of the time, with ≈20% of the NK cells being motile for the whole track (Fig. 3B–D). There could be several reasons for this observed difference between individual NK cells. A strong contribution probably comes from different inherent motility for individual cells, but also local differences in the matrix structure or conjugation to target cells could influence this. Based on the fraction of time spent in TMAPs the tracks were divided into groups with low (<10%), medium (10–90%) and large (>10%) amount of time in TMAPs (Fig. 4). As expected, the NK cells spending most their time in TMAPs were very immotile and confined. Cells in TMAPs were also rounder indicating that they were not actively moving (Supporting Fig. S4A). It is possible that a fraction of these cells was dying. In the group of NK cells spending low or medium amount of time in TMAP individual cells alternated between modes of migration (Fig. 4C,D). To characterize this stop-and-go behavior, we assessed how frequently individual cells in the whole population alternated between modes of migration. As expected, there was a large spread here too, with some cells showing no alternations (primarily immotile cells being scored in a single TMAP for the whole track), some cells switching every 1–3 hours and a few cells switching several times an hour (Fig. 4E). On average cells spent 106 min within a mode of migration before switching. Thus, the analysis of transient migration behavior showed that a majority of NK cells alternated between different modes of migration while a small number of cells were immotile throughout the assay.

### Contact and killing dynamics of NK-target cell interactions in the 3D matrix

**Figure 5:**
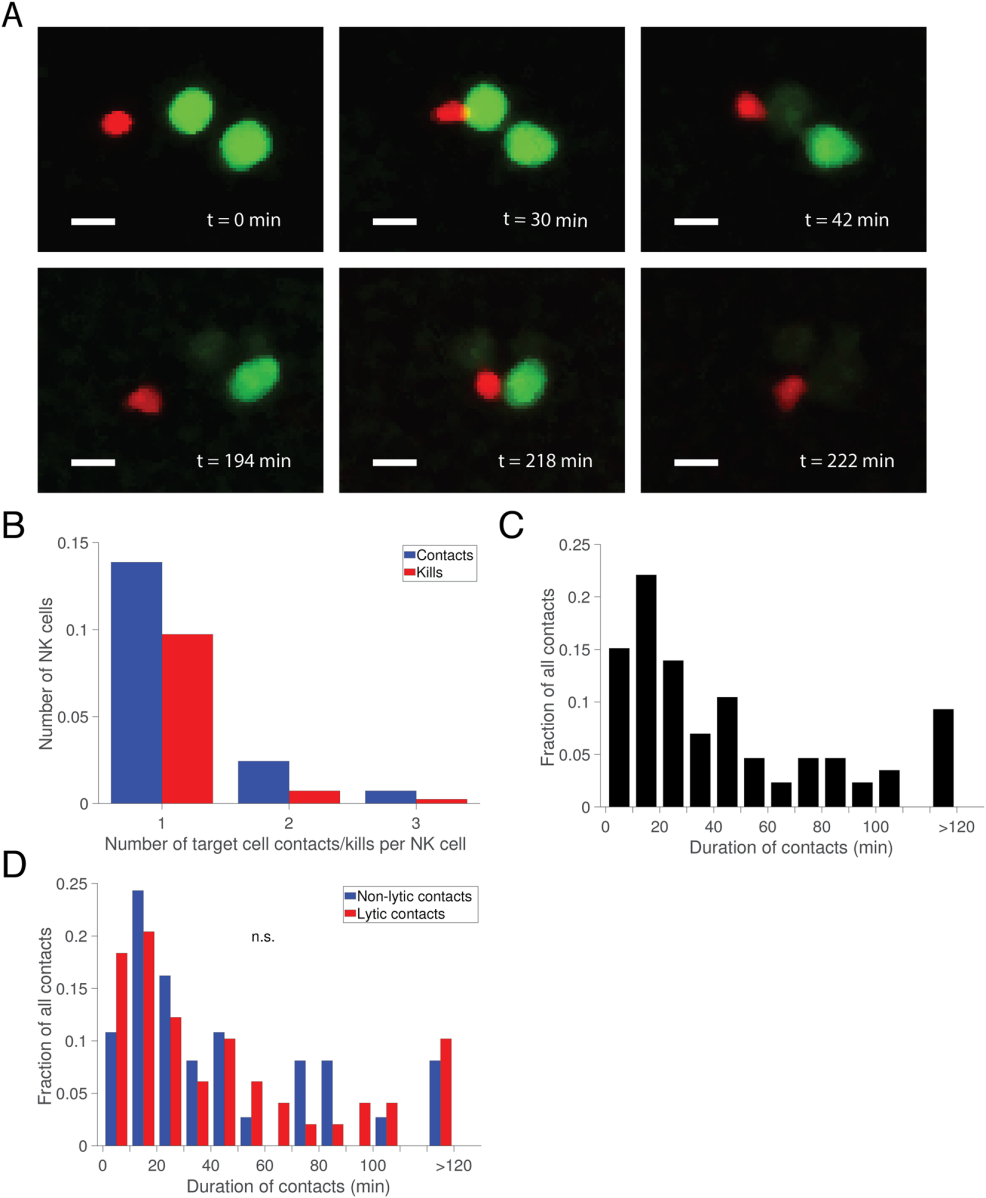
NK cell-mediated lysis of target cells in 3D collagen matrix. (**A**) Time-lapse sequence showing a NK cell migrating and killing two target cells. Scale bar = 15 μm. (**B**) Number of target cell contacts and kills made by individual NK cells. (**C**) Distribution of duration of contact periods (*n* = 86). (**D**) Distribution of duration of contact periods divided by cytolytic and non-cytolytic interactions (*n*_cytolytic_ = 49 and *n*_non-cytolytic_ = 37).

Next, we assessed interaction dynamics between NK and target cells embedded in the matrix. Occasionally migrating NK cells encountered slow-moving target cells in the matrix and were observed to contact and kill target cells (Fig. 5A and Supporting Movies 2 and 3). Contacts between NK and target cells were detected automatically by defining cell-cell contact to occur when the center-to-center distance of NK and target cells were smaller than 20 μm. A large fraction (83%) of tracked NK cells never interacted with target cells in the 3D volume. This number is significantly higher than we observe in typical 2D experiments. Reasons for this could that the nearest neighbor distance is usually greater in this 3D environment compared to a 2D environment and cells migrating randomly in only two spatial dimensions have a higher likelihood of bumping into each other. Indeed, we found that initial distance to the nearest target cell was an important factor for formation of cell-cell contact (Supporting Fig. S5B,C). The migration speed was also slightly higher for NK cells that formed target cell contacts. (Supporting Fig. S5D,E). Out of the 17% NK cells that did interact with targets, some managed to make multiple contacts with a maximum score of 3 contacts (Fig. 5B). Overall, 57% of the NK-target cell contacts lead to target cell death (Fig. 5B). This fraction is similar to what has been previously observed in 2D experiments where ≈20–60% of the conjugates between NK and 293T target cells have led to killing^20^. In the assay we observed NK cells killing up to 3 target cells but this number was most likely restricted by the number of contacts being formed rather than killing capacity.

The mean conjugation period lasted 47.9 ± 49.5 min. (Fig. 5C). This could be compared to the corresponding values for IL-2 activated NK cells in 2D (conjugation time = 65 min). This shorter conjugation time could reflect the importance of mechanical forces alongside biochemical cues when breaking up an NK-target conjugate. In 2D live-cell imaging experiments it is commonly observed that conjugates have a long phase after a conjugation period where the two cells appear to try to break away from each other, sometime leading to target cells piggybacking on migrating NK cells^19,20,28^. Such a phase, which we have previously referred to as attachment, was rarely observed in the 3D matrix. Although similar behavior has been reported also in lymph nodes, the presence of a 3D matrix that offers multiple attachment points might make it easier for two cells to break free from one another^29,30^. We found no significant difference in conjugation time between cytolytic and non-cytolytic contacts (Fig. 5D). Our data shows that NK cells formed conjugates with target cells in the 3D matrix and that these contacts were generally shorter compared to what we observe in 2D. More than half of the NK-target cell conjugates lead to killing with dynamics consistent with NK cell degranulation.

## Discussion

Here we present an assay for analysis of 3D migration and cytotoxicity that allows following a small, spatially confined population of cells for extended periods of time. Imaging in 3D with sensitive detection allows characterization of cell migration, cell-cell contact and killing dynamics. Single cell tracking and detection of cell-cell contacts was automated while accurate determination of target cell death was done by manual analysis.

Depending on cell type and composition of the ECM, cells employ different migration strategies in ECM^31^. Cells expressing lower levels of integrin, e.g. T cells and NK cells, largely migrate using integrin-independent amoeboid-like migration^32^. The studied NK cells were found to exhibit a range of different migration behaviors. Cells were found to alternate between periods of random movement, directed migration and migration arrest. Confocal reflection imaging has shown that most cells encounter pores with average diameters smaller than the typical cell size^23^. Thus, cell locomotion in the 3D matrix requires either that the NK cells accommodate their morphology to the local ECM environment in order to migrate by propulsive squeezing (so-called amoeboid migration), or that they degrade the local matrix by matrix metalloproteinases, and migrate through integrin-dependent, mesenchymal-style migration. In the current analysis, we have not addressed the respective contribution from these mechanisms but it is possible that these factors contribute to the transient migration behavior of the NK cells.

IL-2-activated NK cells were found to form shorter contacts than in comparable 2D assays. A possible mechanism for the shorter contacts could be that cells migrating in a 3D environment have higher density of ECM ligands with which they can bind to generate traction that facilitates breaking up a target cell contact. Our results are consistent with NK cells imaged *in vivo*, where they were shown to make frequent short interactions with target cells, compared to the relatively long contacts established on 2D surfaces^33^.

Target cell density presents an issue when comparing results from 2D and 3D data. To approximate the target cell density for experiments performed in 2D, it is likely that an order of magnitude more target cells are required in the 3D assay. Here we used far lower amounts of cells than that, leading to lower numbers of contacts being recorded. To increase the number of contacts made by individual NK cells the preferred approach would be to increase the number of target cells rather than NK cells. This would likely better represent the situation in a tumor environment but may pose some experimental challenges. Tracking of individual NK cells and detection of cell-cell contacts would still be straight forward, but contacts between individual NK cell and multiple target cells would be more frequent and accurate assessment of target cell death would be more challenging. An automated method for detection of target cell death would be desirable to increase throughput. Current efforts are directed towards dealing with such increased complexity.

The developed microwell-based assay is suitable for 3D time-lapse imaging of NK or T cell migration and cytotoxicity. This system allows for screening of smaller, spatially confined populations of human cells, and can be used to evaluate, e.g. different expansion and activation protocols of cytotoxic cells aimed for applications within cellular immunotherapy.

## Materials and Methods

### NK cells and target cell lines

NK cells were isolated from buffy coats obtained from anonymous healthy donors through the Karolinska Hospital requiring no ethical permit according to local regulations. NK cells were isolated by MACS negative selection according to manufacturer’s instructions (Miltenyi Biotech) and cultured in RPMI supplemented with 10% human serum, 50 U/ml penicillin-streptomycin, 1x non-essential amino acids, 1 mM sodium pyruvate, and 200 U/ml rIL-2 for 5–8 days before used in experiments. The human leukemia K562 cell line was used as target cells. K562 cells were maintained in RPMI 1640 medium supplemented with 10% FBS and 100 U/ml penicillin-streptomycin.

### Fluorescent labeling of NK and target cells

To allow tracking and detection of cytolysis, the K562 target cells were labeled with Calcein green AM (Invitrogen) and the NK cells were labeled with Calcein red-orange (Invitrogen). The cells were washed in PBS before labeling and then incubated in dye-PBS-solution (1 μM) for 10 minutes at 37 °C, washed again and used immediately. Calcein labels the cytosol of viable cells making them clearly detectable by fluorescence microscopy. Upon cell death Calcein green leaks out making it possible to monitor NK cell induced target cell death through loss of fluorescence^34^.

### Hydrogel co-embedding of NK and target cells in microwells

Non-pepsinized rat tail-derived type I collagen was used to reconstitute the ECM-mimicking matrix. A stock solution of collagen monomers (3.0 mg/ml) dissolved in acetic acid was brought to the right concentration by addition of concentrated cell medium (5 × RPMI) and reconstituted by raising to neutral pH by adding an appropriate amount of sodium hydroxide (NaOH). pH neutralization initiates spontaneous fibrillogenesis of type I collagen, why the following steps have to be performed quickly. A cell-collagen mix was achieved by adding a (1:5–10) mixture of NK cells and target cells suspended in RPMI. The mix was then deposited onto the microwell chip where it poured into the wells and set. After incubation (30 min, 37 °C), cell medium was pipetted into the reservoir creating a gentle fluid flow. This normally caused excess gel matrix to detach from the chip so that it could be aspirated with the pipette leaving only collagen-embedded cells inside the wells and not at the top. The chip was then placed on the stage of an inverted microscope and kept under physiological conditions (37 °C, 5% CO_2_) during imaging.

### Imaging

The microwell collagen embedded cells were imaged by confocal microscopy (Zeiss 880 Airyscan) with 10× objective (0.45 N.A.). To balance speed and photobleaching with resolution, the confocal pinhole was set to 1.2 Airy units (42 μm) and the entire well-volume divided into 36–39 optical sections of 512 × 512 pixels each, with a voxel size of ≈ 1.1 × 1.1 × 8 μm^3^. The time it took to image the volume of an individual well was approximately 35 seconds. Up to individual wells were imaged every 2 minutes for 9 h. To investigate if labeling and repeated illumination affected the NK cells negatively, we compared migration of labeled NK cells imaged by confocal microscopy with unlabeled NK cells imaged by phase contrast in a conventional widefield microscope (Zeiss Axio Observer 7, Supporting Fig. S2). The structure of the collagen matrix was assessed by confocal reflection microscopy where the sample was excited by the argon-ion laser (λ = 488 nm) and reflections from collagen fibrils were acquired without discriminating emission filters.

### Automatic tracking using the baxter algorithms

Tracking was performed on 8 wells from 3 independent experiments using the Baxter Algorithms, a MATLAB-based software package for automated cell tracking^15^, which is available for download here (https://github.com/klasma/BaxterAlgorithms). First, the 8-bit images were converted to double images with values between 0 and 1. Then, background artefacts were removed using background subtraction. The background was computed by taking the minimum intensity over time for each voxel position in the *z*-stack. The computed background was then subtracted from the *z*-stacks of all time points in the sequence. The segmentation was performed using Gaussian bandpass filtering followed by thresholding^15,35^. The filter was a difference-of-Gaussians filter where the positive and negative kernels had standard deviations of 1 and 3 pixels respectively. In the *z*-dimension, the standard deviations were divided by a factor of 8, to account for the reduced resolution in that dimension. The threshold level was set to 0.02. To break clusters of cells, a watershed transform was applied to the bandpass filtered image, after performing an *h*-minima transform with an *h*-value of 0.15. Holes in segmented regions were filled and segmented regions with fewer than 5 voxels were removed. Tracks were linked using the global track linking algorithm presented in^16^. The migration scores were computed using a Brownian motion model where the probability distribution of a cells position was a Gaussian distribution centered on the position in the previous time point. The standard deviation of this Gaussian was 6 voxels in the *x*- and *y*-dimensions and 0.5 voxels in the *z*-dimension. In the linking, the probabilities for cell counts in segmented objects were set to 0.2, 0.7, and 0.1, for 0, 1, and 2+ cells, respectively. The probabilities for death and division were both set to 0, and the probability for random appearance and disappearance were both set to 0.001. When the tracks had been created, segmented objects without cells were merged into adjacent segmented objects with cells, to reduce segmentation errors caused by oversegmentation in the watershed transform. To optimize the tracking we used an inbuilt function in the Baxter Algorithms that allow for manual correction of automatically tracked cells. This mainly involves linking broken tracks. Accuracy of the tracking method was assessed by measuring the center-to-center distance of NK cells that had been tracked both manually and automatically (n = 7 cells, n = 1326 tracking points). For a vast majority (94%) of the tracking points the distance was found to be within 2 μm, representing approximately 25% of a cell diameter and 30% of an average step length (Supporting Fig. S6).

### Analysis of migration behavior

Tracks shorter than 1 h and tracks that exhibited a *z* displacement of more than 40 µm in consecutive track positions were excluded from analysis. The mean speed of individual cells was assessed from the displacement between consecutive coordinates in the tracks. To assess confinement we divided the distance between the starting and end coordinates by the total track distance (d_tot_), and this ratio was then corrected for track duration by multiplying with the square root of the total track time (t_tot_) as outlined by Beltman *et al*.^36^.

1 $${\rm{Corrected}}\,{\rm{displacement}}=\frac{\Vert {{\boldsymbol{r}}}_{{\rm{end}}}-{{\boldsymbol{r}}}_{1}\Vert }{{d}_{{\rm{tot}}}}\times \sqrt{{t}_{{\rm{tot}}}}$$

where ${{\boldsymbol{r}}}_{{\rm{end}}}$ is the $(x,y,z)$ position at the end of the trajectory and ${{\boldsymbol{r}}}_{1}$ is the $(x,y,z)$ position at the beginning of the trajectory.

To analyze transient migration behavior in 3D we modified the method previously used to analyze 2D migration^14,20,37^. Briefly, the mean-square displacement in 3D is defined as:

2 $${\rm{MSD}}({t}_{n})=\frac{1}{N-n}(\sum _{i=1}^{N-n}\,{({x}_{i+n}-{x}_{i})}^{2}+{({y}_{i+n}-{y}_{i})}^{2}+{({z}_{i+n}-{z}_{i})}^{2})$$

where N is the number of coordinates, x, y and z the spatial coordinates and t_n_ = n × Δt is the time delay between analyzed coordinates. To study transient variations, the motility coefficient ($M$) was evaluated around a sliding window of N = 25 points for each time point of the trajectory. In 3D, the motility coefficient ($M$) for a random-walk is proportional to the slope of the MSD curve according to:

3 $$MSD=6Mt$$

$M$ was assessed at each time point by linear fitting of the 6 first points of the ${\rm{MSD}}$ curve for each window, giving a M-profile versus time. To determine periods of directed migration we assessed the curvature of the MSD from each time point by estimating the value of *α* in Eq. 4.

4 $${\rm{MSD}}\propto {t}^{\alpha }$$

The value of *α* was obtained by taking the logarithm of Eq. 4 and doing a linear fit to the first 6 points of $\mathrm{log}\,{\rm{MSD}}$ vs $\mathrm{log}\,t$ for each window in the track, giving a *α-*profile versus time. The M and the *α* parameter profiles were then smoothed by rolling average filters before each track was divided into sections of transient migration arrest (TMAP), directed migration and random movement. Parts of trajectories where M was below a set threshold of 4.9 μm^2^/min were classified as in TMAP. The threshold for M was set from a theoretical value obtained for an NK-sized particle (diameter of 8 μm) in Brownian motion^14,20^. Periods where the fitted value of *α* was above 1.5 for at least 10 consecutive time points were defined as periods of directed migration. Parts of trajectories not in TMAP or directed migration were defined as in random movement.

### Evaluation of NK-target cell interaction

NK-target cell contacts were initially detected automatically and defined when the center-to-center distance between an NK and a target cell was less than 20 μm. This distance was determined by manually inspecting images (*n* = 1093) where the center-to-center distance was <30 μm and determining if the cells where in contact or not. With a cut off at 20 μm, 99% of the true contacts were detected (Supporting Fig. S7). The automatically detected contacts were subsequently manually assessed to remove false positive contacts and for determining target cell death by inspection of the fluorescent intensity and integrity of the target cells in individual NK-target cell interactions.

### Statistical analysis

The Mann-Whitney U test was used to evaluate statistical significance. All statistical analysis was performed using MATLAB and p < 0.05 was considered as a significant difference.

## Supplementary information

Supporting figures

Supporting movie 1

Supporting movie 2

Supporting movie 3
