# Supplementary material for: A collagen-based microwell migration assay to study NK-target cell interactions
Source: Sci Rep. 2019 Jul 23;9:10672. doi: 10.1038/s41598-019-46958-3 (PMC6650390; doi:10.1038/s41598-019-46958-3)
Supplement: Supplementary file 1 — Supporting figures [file 41598_2019_46958_MOESM1_ESM.docx]

**A collagen-based microwell migration assay to study NK—target cell interactions**

Per E. Olofsson^1,*^, Ludwig Brandt^1,*^, Klas E. G. Magnusson^2^, Thomas Frisk^1^, Joakim Jaldén^2^ and Björn Önfelt^1,3,4^

^1^Division of Biophysics, Department of Applied Physics, Science for Life Laboratory, KTH Royal Institute of Technology, Tomtebodavägen 23A, 171 65 Stockholm, Sweden

^2^Department of Signal Processing, ACCESS Linnaeus Centre, KTH Royal Institute of Technology, Stockholm, Sweden

^3^Department of Microbiology, Tumor and Cell Biology, Karolinska Institute, Solna, Sweden

^4^Contact: [onfelt@kth.se](mailto:onfelt@kth.se)

* Equal contribution

**Supporting figures**


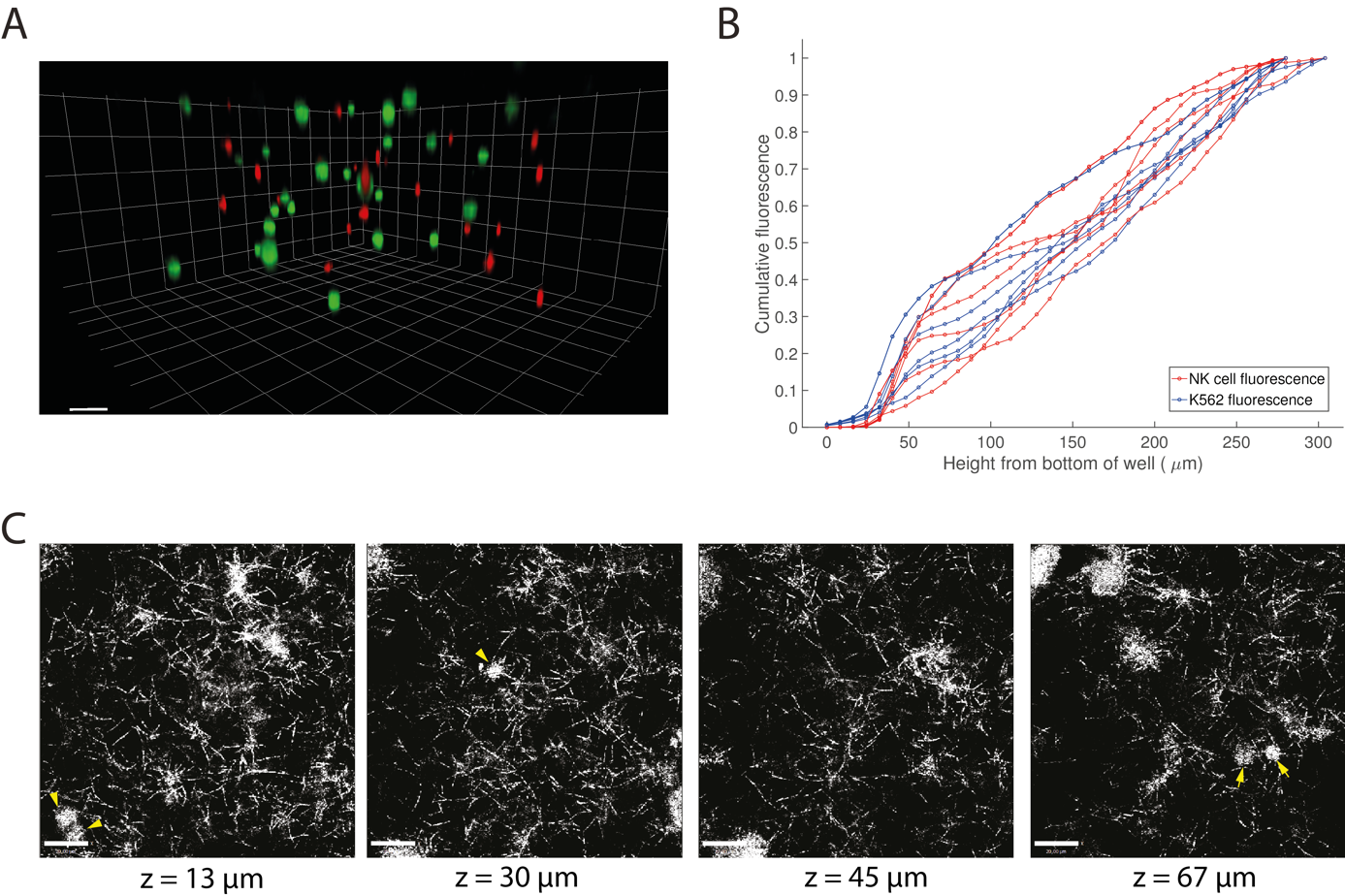


**Figure S1.** Characterization of cellular distribution and matrix structure in microwells. **(A)** Reconstructed 3D view of NK (red) and K562 target cells (green) embedded in collagen matrix. Scale bar = 50 μm. **(B)** Plot of cumulative fluorescence versus distance from the bottom of the microwell indicating the initial distribution of NK cells (red dots) and K562 target cells (blue dots) in the well. **(C)** Confocal reflection microscopy showing optical sections of collagen fibrils at different distances from glass-matrix interface. From left to right: *x-y* plane at *z* = 13 μm, *x-y* plane at *z* = 30 μm, *x-y* plane at *z* = 45 μm, *x-y* plane at *z* = 67 μm. Scale bars = 20 μm. Solid objects (yellow arrowheads) are 10-μm polystyrene beads.

**
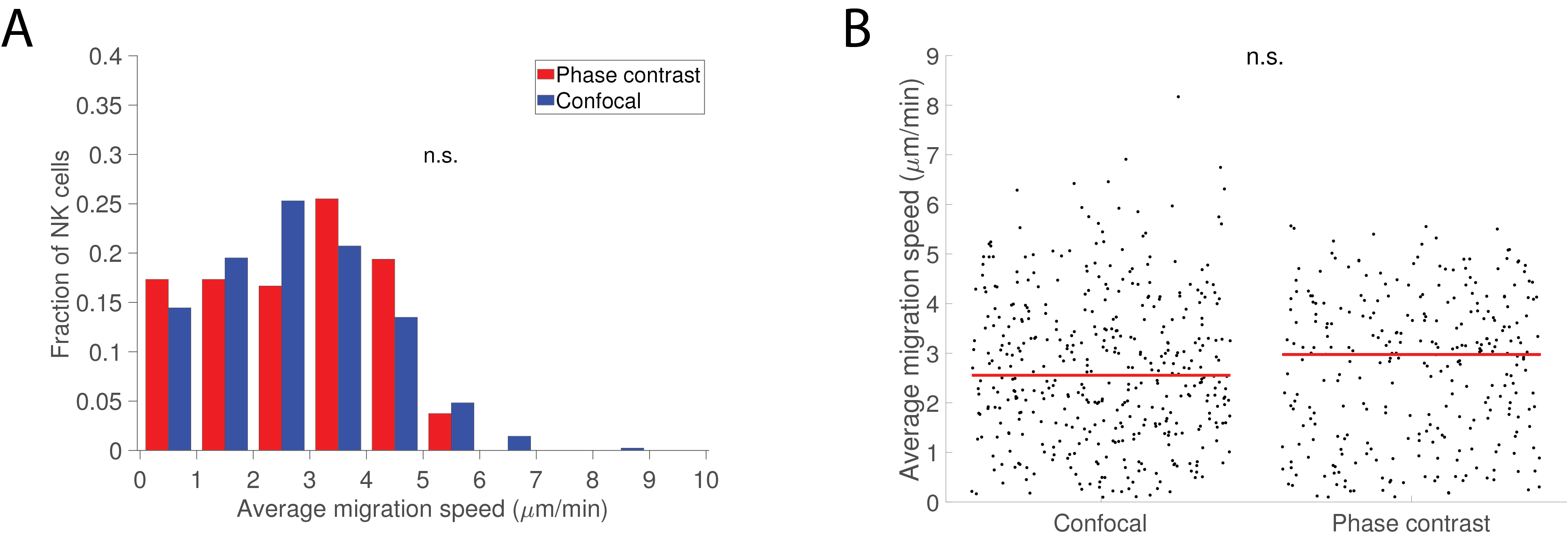
**

**Figure S2.** Distribution of average migration speeds calculated from either Calcein red-orange labeled NK cells imaged using confocal microscopy or unlabeled cells imaged using phase contrast microscopy. The NK cells were imaged alone without target cells in the phase contrast experiments. The automatic tracking was in performed on 2D images created from z-projections of confocal and phase contrast z-stacks, since it was not possible to perform 3D tracking on the phase contrast data. **(A)** Histogram of average migrations speeds measured for cells imaged by confocal (blue) and phase contrast microscopy (red). **(B)** Scatter plot of the same data as in A. Red bars are showing mean values (red bar). Total number of NK cells *n*_confocal_ = 411 and *n*_phase_ = 294.

**
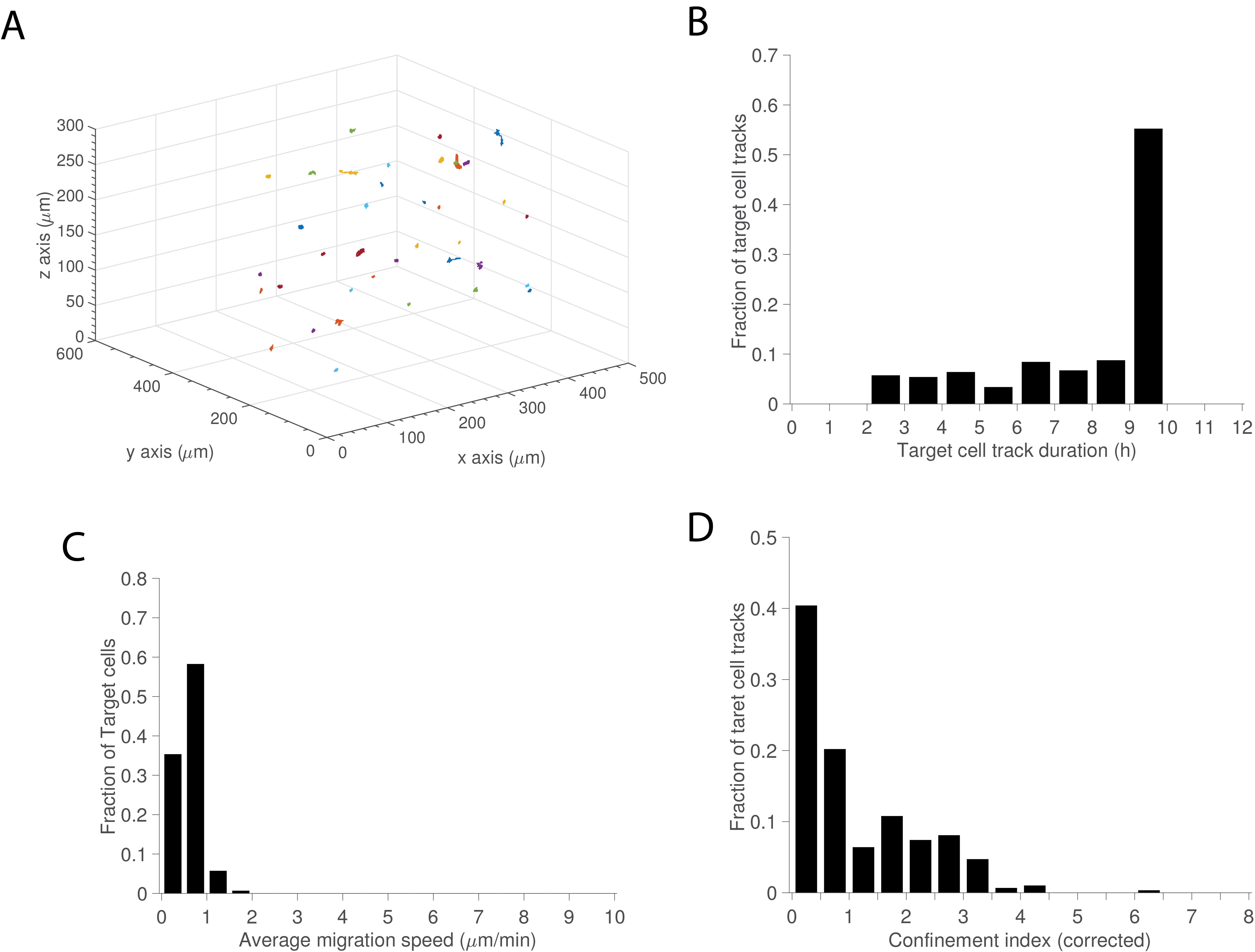
**

**Figure S3.** K562 target cell migration in 3D. **(A)** Track plot of target cell migration trajectories. **(B)** Distribution of target cell track lengths (>1 h). **(C)** Distribution of average migration speeds calculated from individual tracks. **(D)** Corrected confinement index calculated from individual tracks. Total number of K562 target cells *n* = 297.

**
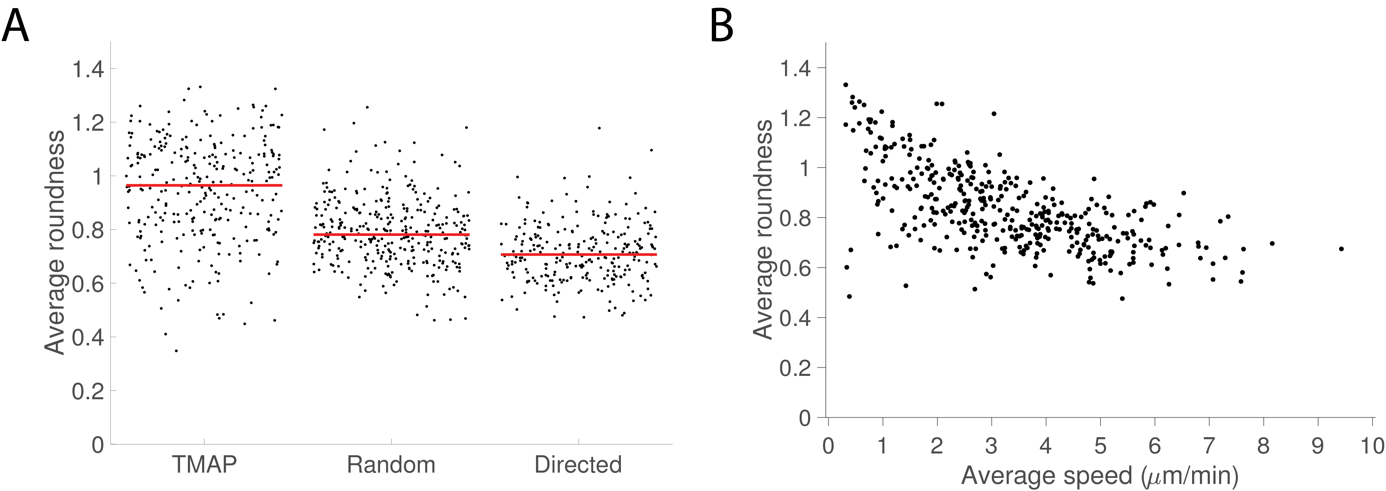
**

**Figure S4.** Roundness of NK cells imaged in the matrix. NK cell roundness was assessed on 2D projections of 3D stacks acquired by confocal microscopy. Roundness was defined as:

$\text{Roundness}= \frac{4\pi\times\mathrm{Area}}{\mathrm{Perimeter}^{2}}$

Where a higher roundness value indicates a rounder shape. **(A)** Scatter plot of roundness of NK cells in each mode of migration. Median values shown as red bars. **(B)** Scatter plot of roundness and average speed. Total number of cells *n* = 396.

**
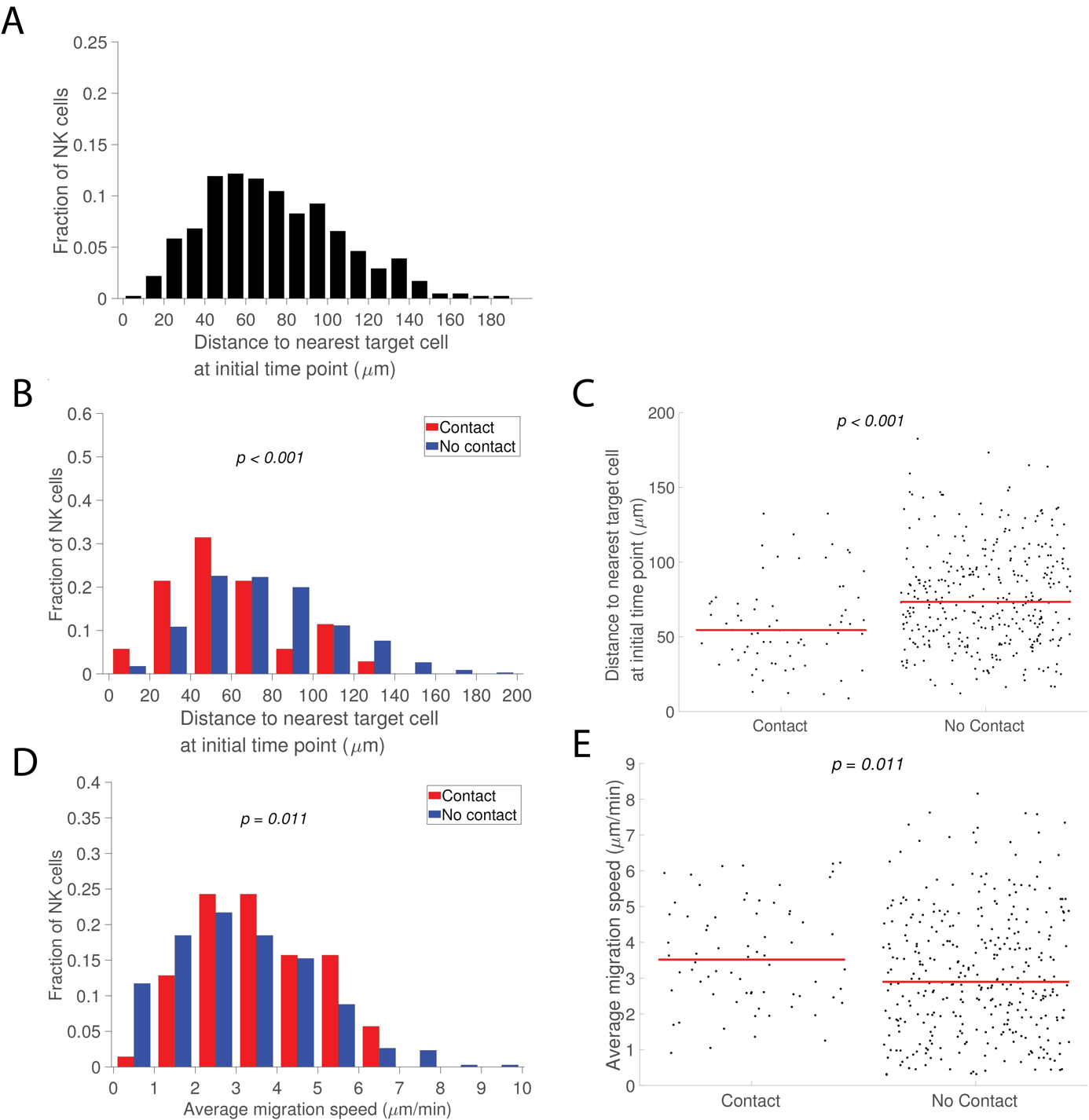
**

**Figure S5.** Assessment of the importance of distance to target cells and migration speed for NK-target cell contacts in the matrix. **(A)** Histogram of distance to the nearest target cell for individual NK cells at the initial time point (n=411). **(B)** Histogram of distance to nearest target cell for individual NK cell at the initial time point, separated into cells forming contacts and no contacts with target cells. **(C)** Scatter plot of the same data as in (B) showing median values as red bars. **(D)** Average migration speed separated into cells forming contacts and no contacts**.** **(E)** Scatter plot of the same data as in (B) showing median values as red bars. Total number of cells forming contacts *n*_contact_ = 70 and not forming contacts *n*_no-contact_ = 341.

**
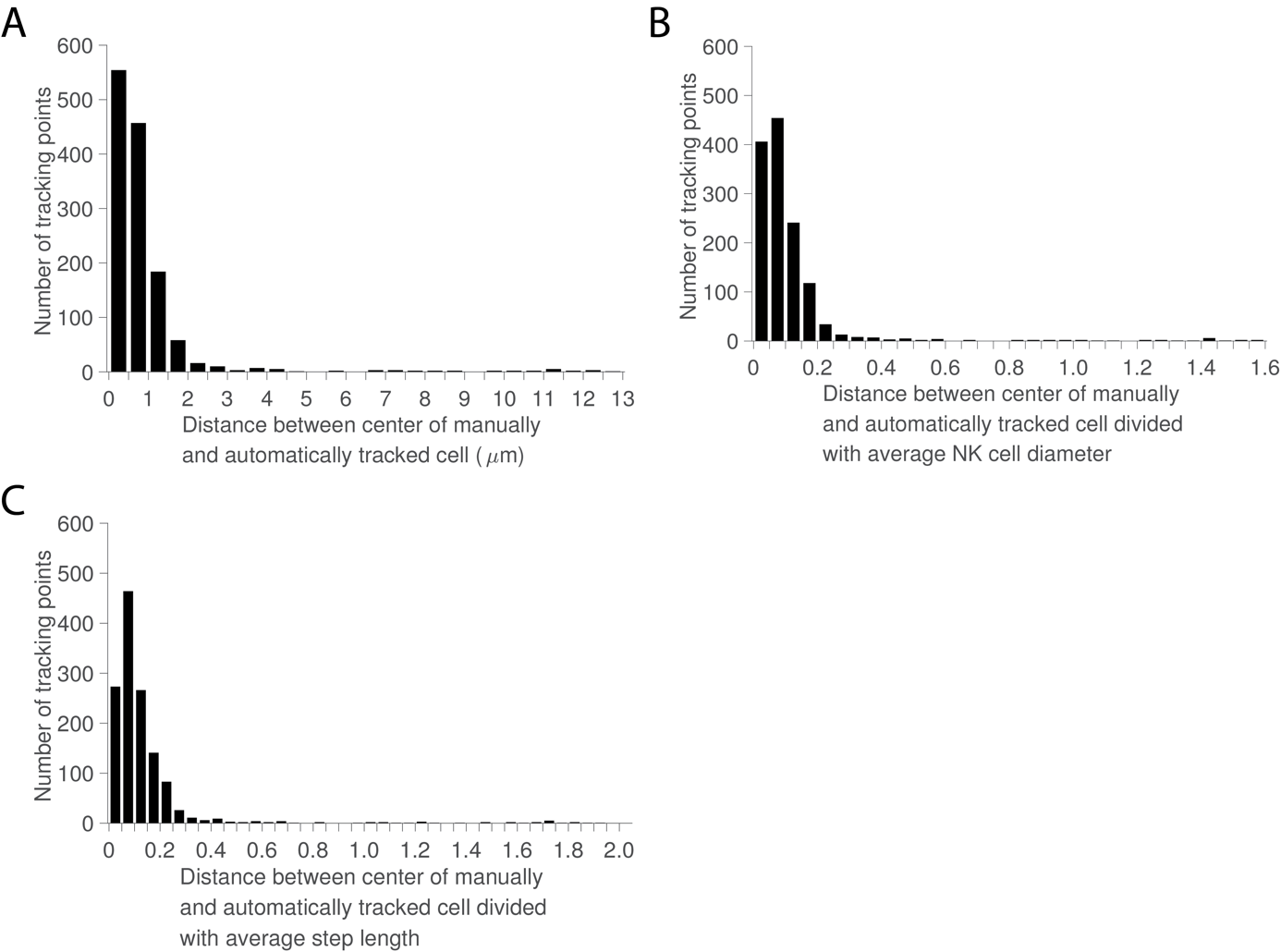
**

**Figure S6.** Error analysis of the automatic tracking. NK cells were tracked both automatically and manually to assess differences in positions determined by the two methods. **(A)** Distance between center of manually and automatically tracked cell. **(B)** Distance between manually and automatically tracked center divided by the average NK cell diameter (8 μm). **(C)** Distance between manually and automatically tracked center divided with the average step length (6.6 μm). Total number of tracking points *n* = 1326. The majority of positions determined by the two methods were within 1 μm from each other, corresponding to differences around 10 % of both NK cell size and step length.

**
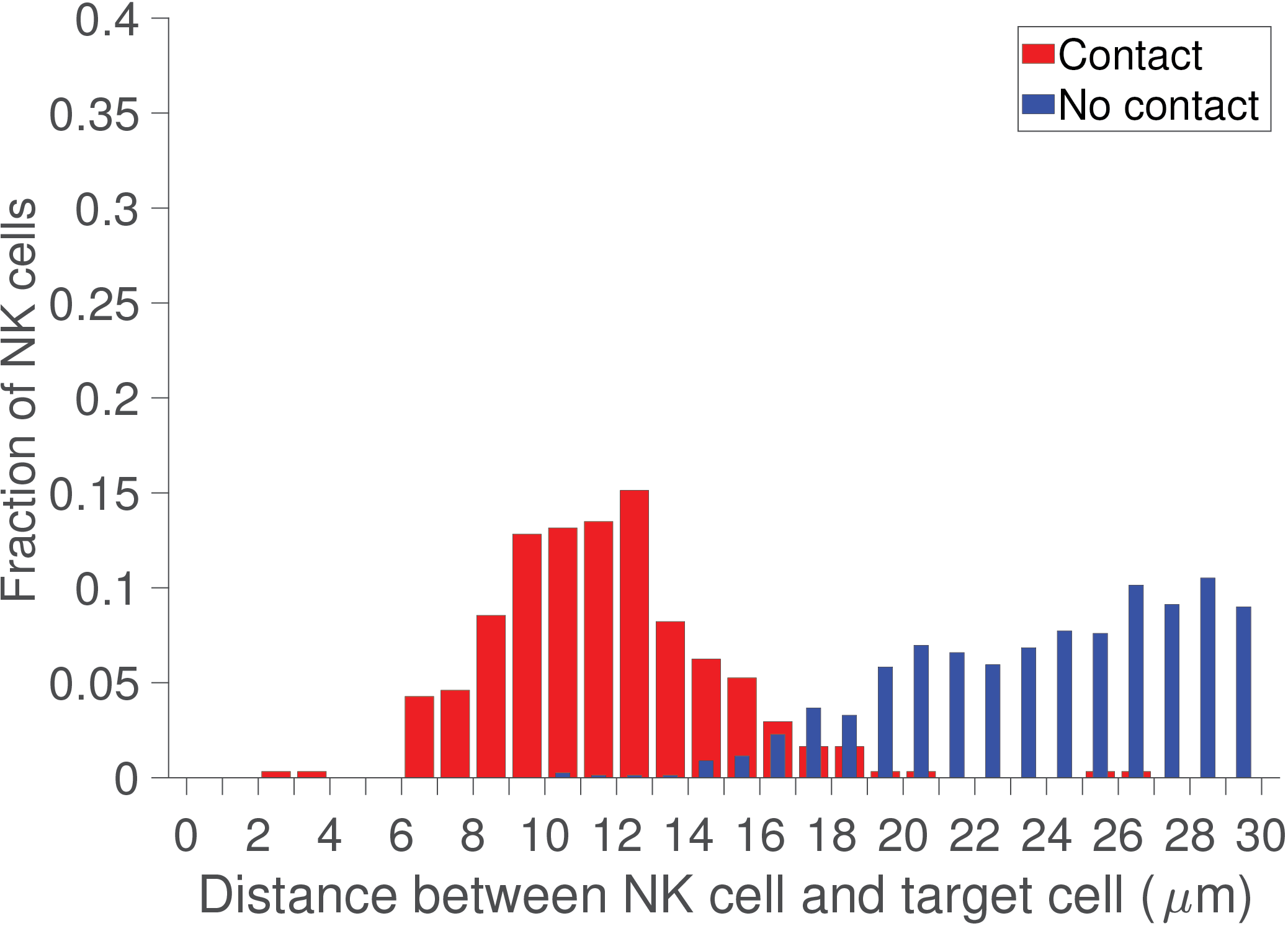
**

**Figure S7.** Manual classification of NK-target cell contacts based on the distance between NK cell and target cells. By automated analysis we detected images (n = 1093) where NK and target cells that had a center-to-center distance of <30 μm and manually determined if NK and target cells were in contact (*n*_contact_ = 304) or not (*n*_non-contact_ = 789). Based on this analysis the threshold of 20 μm used for defining cell-cell contact in the automatic analysis was set.
